# PolyQ Tract Toxicity in SCA1 is Length Dependent in the Absence of CAG Repeat Interruption

**DOI:** 10.3389/fncel.2018.00200

**Published:** 2018-07-31

**Authors:** Suran Nethisinghe, Maria Lucia Pigazzini, Sally Pemble, Mary G. Sweeney, Robyn Labrum, Katarina Manso, David Moore, Jon Warner, Mary B. Davis, Paola Giunti

**Affiliations:** ^1^Ataxia Centre, Department of Molecular Neuroscience, UCL Institute of Neurology, London, United Kingdom; ^2^Neurogenetics Unit, National Hospital for Neurology and Neurosurgery (NHNN), London, United Kingdom; ^3^Molecular Genetics Laboratory, South East Scotland Genetics Service, Western General Hospital, Edinburgh, United Kingdom

**Keywords:** PolyQ, ataxia, CAG repeat, SCA1, neurodegeneration, genetic counseling

## Abstract

Spinocerebellar ataxia type 1 (SCA1) is an autosomal dominant neurodegenerative disorder caused by an expansion of a polyglutamine tract within the *ATXN1* gene. Normal alleles have been reported to range from 6 to 35 repeats, intermediate alleles from 36 to 38 repeats and fully penetrant pathogenic alleles have at least 39 repeats. This distribution was based on relatively few samples and the narrow intermediate range makes the accuracy of the repeat sizing crucial for interpreting and reporting diagnostic tests, which can vary between laboratories. Here, we examine the distribution of 6378 SCA1 chromosomes and identify a very late onset SCA1 family with a fully penetrant uninterrupted pathogenic allele containing 38 repeats. This finding supports the theory that polyQ toxicity is related to the increase of the length of the inherited tracts and not as previously hypothesized to the structural transition occurring above a specific threshold. In addition, the threshold of toxicity shifts to a shorter polyQ length with the increase of the lifespan in SCA1. Furthermore, we show that SCA1 intermediate alleles have a different behavior compared to the other polyglutamine disorders as they do not show reduced penetrance when uninterrupted. Therefore, the pathogenic mechanism in SCA1 is distinct from other cytosine-adenine-guanine (CAG) repeat disorders. Accurately sizing repeats is paramount in precision medicine and can be challenging particularly with borderline alleles. We examined plasmids containing cloned CAG repeat tracts alongside a triplet repeat primed polymerase chain reaction (TP PCR) CAG repeat ladder to improve accuracy in repeat sizing by fragment analysis. This method accurately sizes the repeats irrespective of repeat composition or length. We also improved the model for calculating repeat length from fragment analysis sizing by fragment analyzing 100 cloned repeats of known size. Therefore, we recommend these methods for accurately sizing repeat lengths and restriction enzyme digestion to identify interruptions for interpretation of a given allele’s pathogenicity.

## Introduction

Spinocerebellar ataxia type 1 (SCA1) is an autosomal dominant progressive neurodegenerative disorder resulting in a loss of coordination and balance. SCA1 is characterized by neuronal loss in the cerebellum, brain stem and the spinocerebellar tracts (Greenfield, 1954; Giunti et al., 1994; Zoghbi and Orr, 1995). SCA1 is caused by an expansion of a cytosine-adenine-guanine (CAG) repeat, encoding glutamine, in the gene *ATXN1*, the function of which is still to be determined. Disease onset is typically in the third or fourth decade, but childhood onset has also been reported (Currier et al., 1972; Zoghbi et al., 1988; Schöls et al., 1997). There is an inverse correlation between the CAG repeat length and the age at disease onset that is dramatically improved if one takes into account the longest contiguous CAG repeat stretch in the expanded allele (Orr et al., 1993; Jodice et al., 1994).

Current molecular genetic testing for SCA1 relies on polymerase chain reaction (PCR) of the CAG repeat region using a fluorescently-labeled primer followed by fragment length analysis. Intermediate and borderline alleles of 35–39 repeats are then further analyzed for the presence of interruptions using a restriction enzyme (*Sfa*NI or *Lwe*I). Normal alleles have less than 35 repeats (Ranum et al., 1994; Goldfarb et al., 1996), whilst a subject with 44 repeats has been reported as being asymptomatic at the age of 66 years due to the presence of cytosine-adenine-thymine (CAT) interruptions (Goldfarb et al., 1996). Intermediate alleles have a repeat size of 36–38 repeats with no CAT interruptions. Full penetrance alleles have 39 uninterrupted repeats or greater (Goldfarb et al., 1996; Zühlke et al., 2002). For alleles of 39–44 repeats to be considered pathogenic they must contain an uninterrupted stretch of CAG of at least 39 repeats (Menon et al., 2013). These ranges, however, have been defined using very small sample sizes and therefore there is a need for refining them with a view to precision medicine.

We have previously shown that although there is a very significant correlation between cloned repeat lengths of known size determined by sequencing and their length determined by fragment analysis, the relationship is not identical and there are distinct repeat length differences between the two methods (Menon et al., 2013). The longer the repeat size the greater the difference between the fragment sizing and sequencing methods. An explanation for this size-dependent difference could be the greater GC-content in the longer CAG repeat migrating faster through the POP-7 capillary electrophoresis polymer compared to the commercial 500 LIZ size standards (Menon et al., 2013). In light of our previous study, a correction factor of 3 repeats is now added to the calculated repeat size from fragment analysis in our diagnostic laboratory. This correction factor is suitable for repeat sizes in the intermediate range, but less so for repeat sizes at the extremes.

Triplet repeat primed PCR (TP PCR) is a PCR amplification method using a fluorescently-labeled locus-specific primer flanking the CAG repeat together with paired primers amplifying from multiple priming sites within the CAG repeat itself (Warner et al., 1996). The amplification of the CAG repeats gives a characteristic ladder on the fluorescence trace from a DNA analyzer. This method is particularly useful in detecting large CAG repeat expansions (Warner et al., 1996).

Accuracy in sizing repeats for polyglutamine disorders is critical for precision medicine where a single repeat difference is crucial to determine a diagnosis, particularly for borderline alleles. In addition, the composition of the repeat is crucial for the pathogenesis of the allele.

Here, we screen the largest SCA1 allele cohort to date to explore how the size and composition of the polyQ tract influence the pathogenesis in this disease. We identify a family with a very late age at onset ataxia in which affected subjects carry a pure 38 CAG repeat tract, the lowest pathogenic SCA1 allele reported so far. This is in agreement with the negative correlation between repeat length and age at onset. Interestingly, Klein et al. (2007), using a combination of biochemical and biophysical approaches, compared the structural properties of long and short polyQ tracts under various conditions. They showed that pathogenic and non-pathogenic lengths of CAG repeat studied were soluble species and they displayed similar structural features whilst repeat length only influences the aggregation kinetics and stability. They concluded that the polyQ toxicity does not depend on a structural transition occurring above a specific threshold, but rather that polyQ tracts are toxic sequences, whose deleterious effect gradually increases with their length and that they are lifespan dependent. Our observation of an uninterrupted 38 CAG in a subject developing the condition so late in life (more than 66 years of age) seems to support this basic research observation.

We also determine that the presence or absence of CAT interruption is fundamental in the determination of the pathogenic allele in SCA1. Moreover, we show that a TP PCR-generated CAG repeat ladder can be used to accurately size SCA1 alleles by fragment analysis irrespective of their repeat length or composition. Finally, we improve the model for calculating the number of repeats from fragment analysis sizing values to increase accuracy over a broader range of repeat lengths.

## Materials and Methods

### Ethical Statement

This research has been approved by the London (Queen Square) NHS Research Ethics Committee (reference 09/H0716/53) at the National Hospital for Neurology and Neurosurgery, London.

### Patient Cohort

Blood from patients with an ataxic phenotype were sent to the Neurogenetics Unit at The National Hospital for Neurology and Neurosurgery, London to undergo a panel of diagnostic tests for SCA1, 2, 3, 6 and 7. We selected a cohort of patients who were tested for SCA1 for this study.

### SCA1 Fragment Sizing and Cloning of SCA1 Allele CAG-Repeat Tracts

Genomic DNA was extracted from patient peripheral blood leukocytes using a FlexiGene DNA kit (QIAGEN). SCA1 alleles were fragment sized as previously described, amplifying the CAG-repeat tracts with a FAM-labeled PCR primer and resolving fragments on an ABI 3730*xl* DNA analyzer with a GeneScan 500 LIZ Size Standard (Thermo Fisher Scientific; Menon et al., 2013). Repeat lengths are calculated by subtracting the number of extraneous bases in the PCR product outside of the repeat region (139 bp) and then dividing by 3. A correction factor of three repeats is then added, based on the difference previously seen between fragment analyzed and sequenced clones (Menon et al., 2013). For alleles with 35–39 repeats, PCR products were digested with *Sfa*NI. This restriction enzyme cuts the PCR product if a CAT interruption is present and allows the distinction of large normal (interrupted) and pathogenic (uninterrupted) alleles. SCA1 allele CAG-repeat tracts were cloned into a pcDNA3.1(+) vector as previously described (Menon et al., 2013) or pCR-Blunt using the Zero Blunt PCR Cloning kit (Thermo Fisher Scientific) and propagated in Stbl3 *E. coli*.

### Triplet Repeat Primed PCR (TP PCR)

TP PCR was performed with plasmids carrying cloned CAG-repeat tracts using a primer mix P1, P3 and P4CTG according to protocols previously described (Warner et al., 1996).

## Results

### SCA1 Allele Distribution in a Large UK Cohort

**Figure 1:**
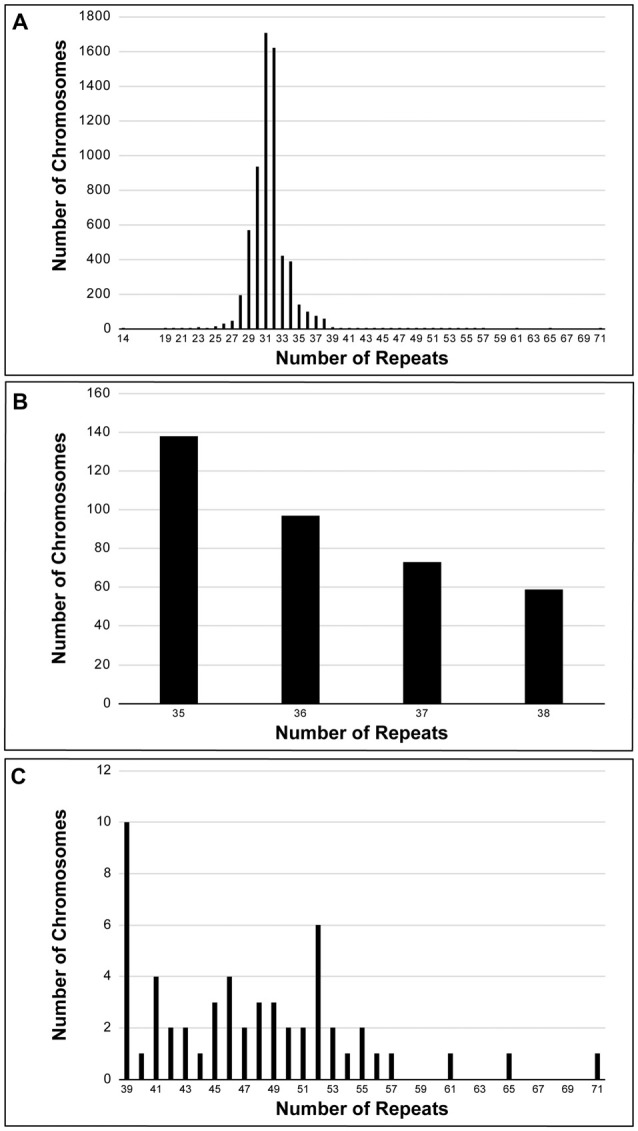
Frequency distribution of spinocerebellar ataxia type 1 (SCA1) alleles performed on a UK cohort at the Neurogenetics Unit, National Hospital for Neurology and Neurosurgery, London. Six-thousand three-hundred and seventy-eight discrete chromosomes were analyzed for SCA1 **(A)**. The alleles are normally distributed with the most frequent allele having 31 repeats. Three-hundred and sixty-seven chromosomes were sized in the intermediate range (35–38 repeats) **(B)**, whilst there were 54 chromosomes in the pathogenic range (39–71 repeats) **(C)**. All intermediate and expanded alleles were digested with *Sfa*NI to identify interruptions. Only one allele (0.3% of intermediate alleles) was uninterrupted and had 38 repeats. The most frequent pathogenic allele had 39 repeats (*n* = 10) with the next most frequent allele having 52 repeats (*n* = 6).

SCA1 diagnostic tests were performed on 6378 discrete chromosomes by the Neurogenetics Unit at The National Hospital for Neurology and Neurosurgery, London. The frequency and distribution of the repeat sizes within the cohort can be seen in Figure 1A, with an enlarged view of alleles in the intermediate range (35–38 repeats) shown in Figure 1B and expanded alleles (39–71 repeats) shown in Figure 1C. The data are normally distributed with the majority of chromosomes having normal alleles in the range 14–34 repeats (*n* = 5957). Pathogenic alleles in the cohort range from 39 to 71 repeats, with this range being extended by single individuals with 65 and 71 repeats (*n* = 54). The most frequent pathogenic allele had 39 repeats (*n* = 10) with the next most frequent allele having 52 repeats (*n* = 6). Age at onset data was available for 44 individuals with an uninterrupted pathogenic allele, which shows a significant negative correlation with repeat length (Pearson correlation coefficient *r* = −0.7751, significant at the 0.0001 level; Supplementary Figure S1). Other SCA diagnostic tests for these individuals were in the normal range (mean ± standard deviation)–SCA2 (22 ± 1 repeats, *n* = 18), SCA3 (26 ± 6 repeats, *n* = 18), SCA6 (12 ± 1 repeats, *n* = 19), and SCA7 (12 ± 1 repeats, *n* = 18). There were 367 chromosomes that fall within the intermediate range (35–38 repeats) and out of these SCA1 diagnostic tests only one repeat PCR product was resistant to digestion by the restriction enzyme *Sfa*NI. This suggests that this allele was pathogenic and uninterrupted.

### Clinical Presentation of Family With Lowest Number of Pathogenic CAG Repeats

The proband from a family of Indian Asian origin presented with a phenotype consistent with autosomal dominant cerebellar ataxia (ADCA) type I (Harding Classification; Harding, 1982) and an age at onset of in her mid 60s (II:3, Figure 2A). The proband’s mother (I:2) had a progressive ataxia balance problem with an onset around 70 years and was still walking around her house cruising furniture, prior to her death at 79 years old. The proband’s older sister (II:2) had slurred speech and progressive balance disorder from about the age of 60 years old and walked with a Zimmer frame. She died of stomach cancer at the age of 82 years old.

**Figure 2:**
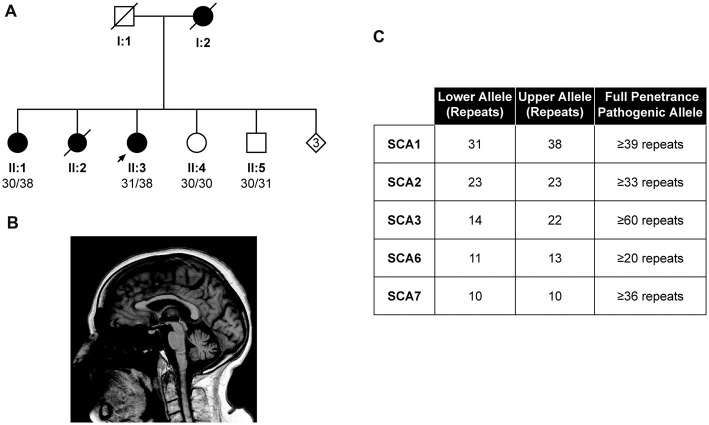
Identification of the lowest reported SCA1 pathogenic allele to date. Pedigree showing the proband (II:3) and affected individuals (I:2, II:1 and II:2) with a autosomal dominant cerebellar ataxia (ADCA) type 1 phenotype **(A)**. DNA was available from individuals II:1, II:3, II:4 and II:5, with the pathogenic allele being sized at 38 repeats in both affected individuals (II:1 and II:3). Sagittal 1.5T T1-weighted brain magnetic resonance imaging (MRI) of the Proband’s eldest sister (II:1) at 88 years old, showing mild midline cerebellar atrophy with the volume of medulla and pons preserved **(B)**. Other diagnostic tests for the proband (II:3) proved negative, suggesting the SCA1 allele of 38 repeats is pathogenic **(C)**.

Magnetic resonance imaging (MRI) of the proband at 66 years old showed global atrophic changes with disproportionate atrophy in the posterior fossa with atrophy of the cerebellum (particularly the vermis), pons and medulla. This pattern is consistent with spinocerebellar degeneration (Döhlinger et al., 2008). A second MRI of the whole spine when she was 73 years old showed nothing of note apart from slight foraminal narrowing at various levels, mostly in the cervical spine, but without compression of the cord and changes in thoracic and lumbar spine consistent with her age. Her cranial nerves appeared to be quite intact without any abnormality. On examination at the age of 75 years old, she showed severe truncal and limb ataxia with dysarthria with limitation of the external ocular movement in the lateral and vertical gaze. Tendon reflexes were normal and absent ankle jerks with extensor plantar. There was severe deep sensory loss bilaterally. Her SARA score at this time was 33.5 (Schmitz-Hübsch et al., 2006).

The proband’s eldest sister (II:1) experienced a slowly progressive balance disorder with a history of falls at the age of 83. She subsequently was investigated and had a laminectomy and excision of an intradural meningioma. She recovered with some improvement however after few months she got progressively worse.

On examination at the age of 88 years old, she showed severe truncal ataxia, brisk reflexes in the upper and lower limbs with mute plantar. Deep sensory impairment was also reported. Brain MRI at this age showed mild midline cerebellar atrophy with the volume of the medulla and pons preserved (Figure 2B). SARA score was 16.5, progressing to 24 after 21 months follow-up, consistent with SCA1 progression (Jacobi et al., 2015).

Fragment sizing analysis of the proband revealed a pathogenic SCA1 allele size of 38 repeats, which could not be digested with *Sfa*NI, whilst she tested negative for SCA2, 3, 6 and 7 (Figure 2C). Exome sequencing of the index case revealed no pathogenic mutations in the genes of the Hereditary Ataxia Panel at Genomics England, suggesting the 38 repeat allele is pathogenic (data not shown). The alleles from this patient were cloned and sequenced, which confirmed a mean pathogenic allele size of 38 repeats (Supplementary Table S1). Subsequent fragment sizing analysis of the proband’s siblings revealed her eldest sister (II:1) was affected, whilst her younger siblings (II:4 and II:5, Figure 2A) were unaffected.

### Triplet Repeat Primed PCR (TP PCR) CAG Repeat Ladder Accurately Sizes Uninterrupted and Interrupted Repeats

**Figure 3:**
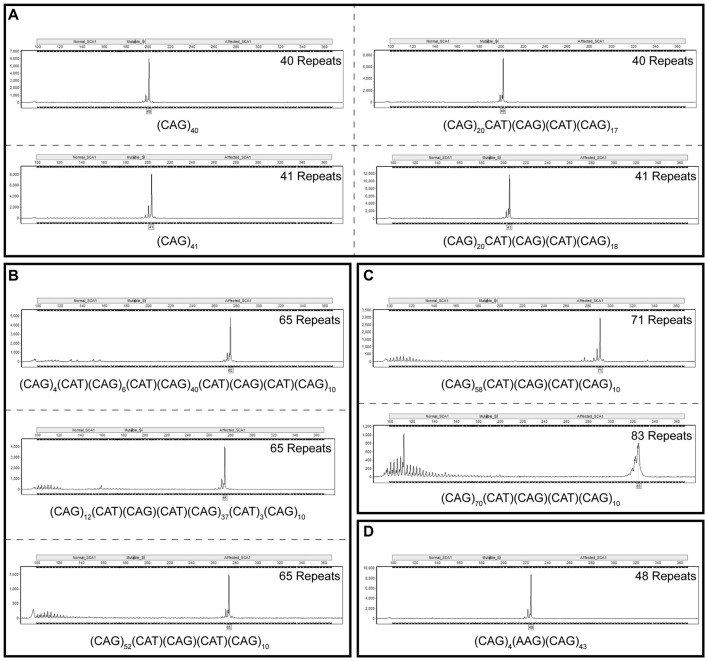
Fragment analysis alongside a triplet repeat primed polymerase chain reaction (TP PCR) cytosine-adenine-guanine (CAG) repeat ladder accurately sizes cloned SCA1 alleles irrespective of repeat sequence or length. Fragment analysis traces for cloned SCA1 alleles of 40 and 41 repeats, with and without cytosine-adenine-thymine (CAT) interruptions **(A)**. Fragment analysis traces for cloned SCA1 alleles of 65 repeats with different interruption configurations **(B)**. Fragment analysis traces for long, interrupted SCA1 alleles with total lengths of 71 and 83 repeats **(C)**. Fragment analysis trace for a SCA1 allele with a point mutation resulting in an atypical AAG interruption **(D)**. Individual fragment analysis traces are available as Supplementary Material (Supplementary Datasheet S1).

In order to take into account for the distinct migration pattern of CAG repeats by fragment analysis capillary electrophoresis, TP PCR CAG repeats were used as a ladder to improve the accuracy of sizing CAG repeats by fragment analysis. Plasmids containing cloned repeat configurations of known size and sequence were analyzed in a linked anonymous manner by fragment analysis alongside a TP PCR-generated CAG repeat ladder. Repeat sizes determined through this method match sizes determined by sequencing the plasmids. Figure 3A shows TP PCR reads for two plasmids with 40 repeats and two plasmids with 41 repeats, with and without interruptions. Plasmids with 65 repeats and three different interruption configurations are shown in Figure 3B. Each of these configurations are accurately sized as having 65 repeats by TP PCR. Figure 3C shows TP PCR reads for two of the larger interrupted repeats of 71 and 83 repeats, respectively. A plasmid with 48 repeats and a unique AAG interruption is also accurately sized by TP PCR (Figure 3D).

### Improving the Accuracy of Determining Repeat Number From Fragment Analysis Data Using a Model From Plasmids Containing Known CAG Repeat Tract Sequences

**Figure 4:**
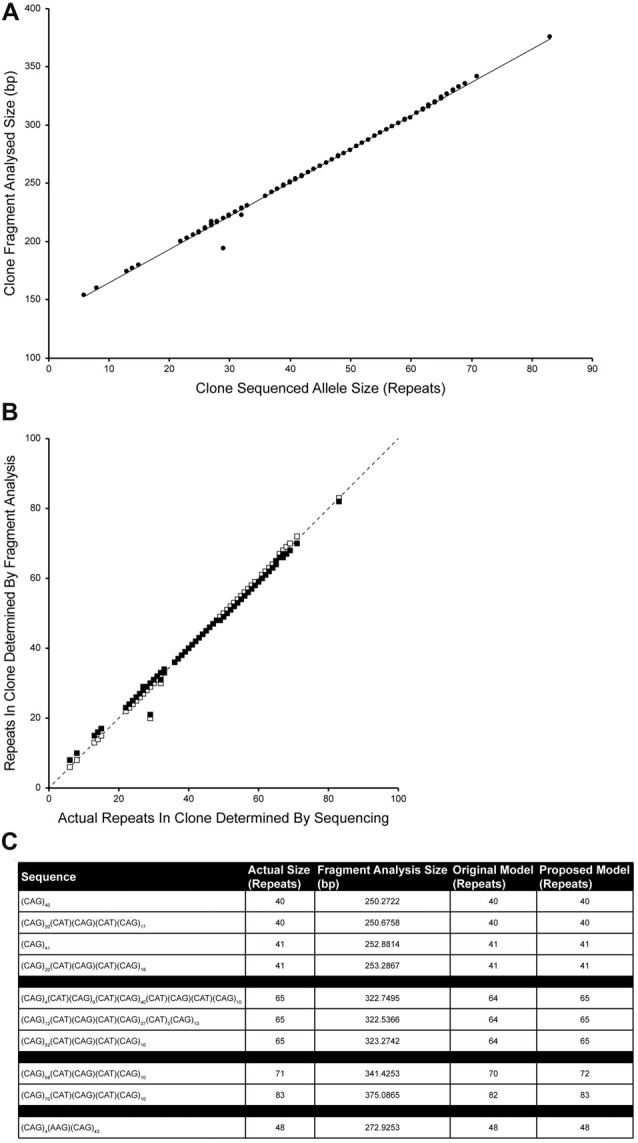
Improving the accuracy of calculating repeat length from fragment analysis size data. One-hundred cloned SCA1 alleles of known sequence were used as templates for fragment analysis by capillary electrophoresis. The fragment analyzed size was plotted against the actual size of the CAG repeat determined by sequencing **(A)**. The data tightly fit a linear model (Number of repeats = [(Fragment analyzed size − 135.8487)/2.8669]; *R*^2^ = 0.99675), with one significant outlier. Comparing the proposed model (white squares) with the original model (Number of repeats = [(Fragment analyzed size − 139)/3] + 3; black squares), the proposed model points fall much closer to the identity line suggesting a better representation of the sequenced repeat lengths by the fragment sizing data **(B)**. To validate the model, plasmids that were previously sized alongside a TP PCR CAG repeat ladder (Figure 3) were fragment analyzed and the number of repeats calculated using either the original model or the proposed model **(C)**. Both models are able to accurately size clones in the range 40–48 repeats, however the proposed model can accurately size clones of 65 and 83 repeats.

The CAG repeat tracts from SCA1 alleles were previously cloned and sequenced (Menon et al., 2013). Fragment analysis was performed on 100 of these plasmids containing CAG repeat tracts of known sequence and length in order to determine an improved relationship between the raw size data from fragment analysis and the actual length of the CAG repeat tract. A new linear model was determined which takes into account the migration of the CAG repeat fragments through the capillary electrophoresis polymer relative to the GeneScan 500 LIZ standards over the most likely repeat ranges to be tested (Figure 4A). The proposed model involves subtracting 135.8487 from the fragment analyzed size, dividing this by 2.8669 and rounding this value to the nearest repeat. The data fits tightly to the proposed model with one significant outlier (*R*^2^ = 0.99675).

Comparing the accuracy of the original (black squares) and proposed models (white squares) in determining the actual number of repeats, the proposed model points fall much closer to the identity line than the old model (Figure 4B).

To validate the accuracy of the proposed model, plasmids that were previously used in sizing alongside a TP PCR CAG repeat ladder (Figure 3), fragment analysis was performed and number of repeats were calculated using original and proposed models (Figure 4C). Both models accurately size repeats of 40, 41 and 48 repeats, however the proposed model also accurately sizes the plasmids with 65 repeats and 83 repeats.

## Discussion

This study presents the largest cohort of SCA1 diagnostic tests reported to date. The alleles from this UK cohort are normally distributed, with the most frequent allele containing 31 repeats. There were 54 SCA1 positive tests in this cohort, which comprise 1.7% of the SCA1 tests performed. Nineteen percent of these positive tests are close to the pathogenic cut-off at 39 repeats. The next most frequent pathogenic allele, comprising 11% of the positive tests, had 52 repeats. Expanded alleles (39–71 repeats) had a mean allele size of 47 ± 7 repeats (mean ± standard deviation) and normal alleles (14–34 repeats) had 31 ± 2 repeats, which is consistent with allele sizes observed in the EUROSCA and RISCA cohorts (Tezenas du Montcel et al., 2014a,b), although these were comprised of fewer SCA1 tests. Interestingly, out of the 367 alleles that fall in the intermediate range (35–38 repeats), only one with 38 repeats (0.3%) was uninterrupted, as determined by resistance to *Sfa*NI restriction enzyme digestion. Alleles with 35–37 repeats (*n* = 308) and the remaining 38 repeat alleles (*n* = 58) have interruptions that bring the contiguous CAG repeat length well below the pathogenic threshold and should not form aggregates according to our cellular model (Menon et al., 2013). Although this data would suggest the normal range should be 6–38 repeats when interrupted, we cannot rule out that the intermediate alleles of 35–38 repeats may form a reservoir for pathogenic alleles since we have previously seen that interruptions can be lost and expansion can occur from one generation to the next (Menon et al., 2013). The traditional threshold should not be viewed as a strict cut off corresponding to the structural toxic threshold proposed by Perutz et al. (2002), but a linear accumulation of aggregates over time (Klein et al., 2007). Indeed, we have observed a very late onset of SCA1 at the age of 66 years old in an individual with 38 repeats. Even polyglutamine peptides as short as 20 repeats can form aggregates and cause cell death when translocated to the nucleus (Yang et al., 2002). Indeed, patients with SCA6 have as few as 20 CAG repeats in the CACNA1A gene (Jodice et al., 1997), indicating that aggregates can form, but protein context is key. In addition, it is interesting to note that the vast majority of these patients have an age at onset typically later than their mid-50s.

Here, we present a family where the proband has the shortest number of CAG repeats associated with an ADCA type I phenotype reported to date. The CAG repeat region was uninterrupted as determined by lack of *Sfa*NI digestion and confirmed by cloning and sequencing of the expanded allele. The pathogenic allele was 38 repeats and segregates with the disease in this family since it was also inherited by the affected eldest sibling, but not the two unaffected siblings. Since this allele appears fully penetrant, we would suggest a reduction in the pathogenic range to incorporate this 38-repeat allele if uninterrupted. With this being the case, and the fact that the remaining intermediate alleles can be digested with *Sfa*NI therefore being classified as normal, then SCA1 now is distinct to the majority of other polyglutamine diseases for which intermediate alleles with reduced penetrance have been reported.

As previously suggested, accuracy of CAG repeat sizing by fragment length analysis may be impaired by the GC-rich, secondary structures associated with the CAG repeat region compared to the GeneScan 500 LIZ sizing standard used (Menon et al., 2013). The longer the repeat region the more compact the secondary structures that are formed and hence the faster the migration through the polyacrylamide matrix in the capillary electrophoresis DNA analyzer. The European Molecular Genetics Quality Network (EMQN) have also proposed that sizing by an internal size marker, such as GeneScan 500 LIZ, may not be accurate since the conversion between fragment size and number of repeats is not necessarily linear (Sequeiros et al., 2010a,b). At that EMQN Best Practice meeting, it was strongly recommended that laboratories testing SCAs in a diagnostic setting use accurate size controls, determined by sequencing (or any other appropriate method), to construct allelic ladders to improve repeatability and reproducibility (Sequeiros et al., 2010b). To this end, we analyzed a panel of clones of known sequence with varying sequence length and repeat configurations by fragment analysis alongside a TP PCR-generated CAG repeat ladder. This method was able to accurately size the repeat region of these cloned SCA1 alleles irrespective of their repeat length or composition. The benefit of TP PCR-generated CAG repeat ladders in a diagnostic setting is clearly demonstrated through the accurate sizing of longer repeats, which are not accurately determined by fragment analysis alone.

To see whether we could improve interpretation of fragment analysis results without the need for running a TP PCR-generated CAG-repeat ladder, we fragment analyzed 100 clones of known sequence that we previously generated (Menon et al., 2013). Since the repeat lengths and structures were known from the plasmid sequencing information, a standard curve or model could be drawn up relating raw fragment analysis size to actual repeat length. The data fit the proposed linear model very tightly and improve the accuracy of determining the repeat size from the raw fragment analysis data. This proposed model provides a more representative relationship between the fragment length and true number of CAG repeats contained therein.

In light of this study, we would recommend diagnostic labs to use either a TP PCR-generated CAG repeat ladder or a series of CAG repeat clone standards to calibrate repeat size calculation from capillary electrophoresis fragment migration. This ideally would be performed with each batch of polymer used for capillary electrophoresis. Further studies would be required to validate variability of results across multiple diagnostic centers. Ultimately, however, cloning and sequencing of the repeat region for each allele is the only way to truly determine the repeat configuration and accurately size the repeat region.

In summary, the identification for the first time of a SCA1 family with a very late onset with only 38 CAG repeats, support the hypothesis that polyQ tracts are toxic in a length and time dependent way (Klein et al., 2007). We have redefined the normal and pathogenic allele ranges based on the presence or absence of interruption, using data from a large cohort. Moreover, we have shown that the intermediate alleles in SCA1 do not exhibit reduced penetrance, as is the case with the majority of other polyglutamine diseases, which makes it unique in the pathogenesis of the disease. Finally, we proposed a method for which the precise number of CAG repeats can be identified using a combination of cloning and the use of a ladder containing CAG repeats of known lengths for sizing. This work is important for refining the methods of sizing SCA1 alleles used in combination with restriction enzyme towards improving the precision of diagnosing ataxic patients and enriching their genetic counseling.

## Author Contributions

SN, MS, RL, JW, MD and PG conceived and designed the experiments. SN, MLP, SP, KM, DM and JW performed the experiments. SN, MLP, SP, MS, RL, DM, JW, MD and PG analyzed the data. SN, RL, JW, MD and PG wrote and revised the manuscript.

## Conflict of Interest Statement

The authors declare that the research was conducted in the absence of any commercial or financial relationships that could be construed as a potential conflict of interest.

## Supplementary Material

FIGURE S1 Correlation between pathogenic SCA1 allele repeat size and age at disease onset. Age at disease onset data was available for 44 individuals with pathogenic SCA1 alleles that were uninterrupted. There is a significant negative correlation (Pearson correlation coefficient *r* = −0.7751, *P* < 0.0001) and the data fit a linear regression model (*y* = −1.494x + 110.6, *R*^2^ = 0.6008). The solid line depicts the fit result while the dashed lines show the prediction bounds at the 95% confidence level.

TABLE S1 Frequency of each SCA1 clone sequence identified for the proband (II:3). *ATXN1* CAG repeats were PCR amplified, cloned and sequenced. Mean allele sizes are 30 repeats and 38 repeats.

DATASHEET S1 Individual fragment analysis traces as shown in Figure 3.

## Abbreviations

ADCA: autosomal dominant cerebellar ataxia
CAG: cytosine-adenine-guanine
CAT: cytosine-adenine-thymine
EMQN: european molecular genetics quality network
MRI: magnetic resonance imaging
PCR: polymerase chain reaction
SCA: spinocerebellar ataxia
TP PCR: triplet repeat primed PCR.
