# Supplementary material for: PolyQ Tract Toxicity in SCA1 is Length Dependent in the Absence of CAG Repeat Interruption
Source: Front Cell Neurosci. 2018 Jul 31;12:200. doi: 10.3389/fncel.2018.00200 (PMC6080413; doi:10.3389/fncel.2018.00200)
Supplement: FIGURE S1 — Correlation between pathogenic SCA1 allele repeat size and age at disease onset. Age at disease onset data was available for 44 individuals with pathogenic SCA1 alleles that were uninterrupted. There is a significant negative correlation (Pearson correlation coefficient r = −0.7751, P < 0.0001) and the data fit a linear regression model (y = −1.494x + 110.6, R2 = 0.6008). The solid line depicts the fit result while the dashed lines show the prediction bounds at the 95% confidence level. [file Table_1.PDF]

Repeat Size	Pattern	Proband (II:3)
27	$(CAG)_{27}$	1
29	$(CAG)_{12}(CAT)(CAG)(CAT)(CAG)_{14}$	1
30	$(CAG)_{12}(CAT)(CAG)(CAT)(CAG)_{15}$	6
32	$(CAG)_{12}(CAT)(CAG)(CAT)(CAG)_{17}$	1
37	$(CAG)_{37}$	3
38	$(CAG)_{38}$	5
39	$(CAG)_{39}$	3
	**Total**	**20**
