# Supplementary figures and images for: PolyQ Tract Toxicity in SCA1 is Length Dependent in the Absence of CAG Repeat Interruption
Source: Front Cell Neurosci. 2018 Jul 31;12:200. doi: 10.3389/fncel.2018.00200 (PMC6080413; doi:10.3389/fncel.2018.00200)

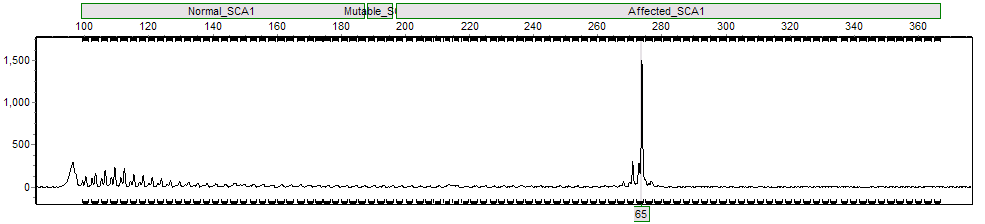

Supplement: TABLE S1 — Frequency of each SCA1 clone sequence identified for the proband (II:3). ATXN1 CAG repeats were PCR amplified, cloned and sequenced. Mean allele sizes are 30 repeats and 38 repeats. [file Data_Sheet_1.zip › Figure 3/Panel B/9966_74147_NoFam_SCA1_E03.png]

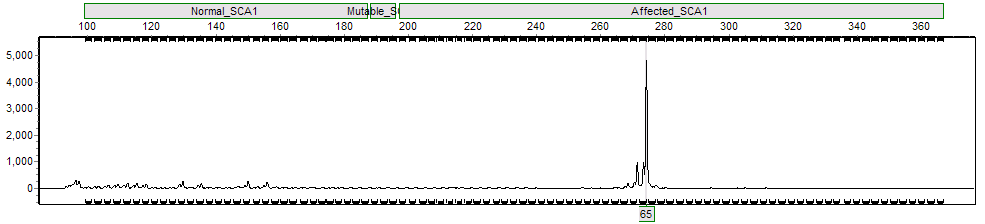

Supplement: TABLE S1 — Frequency of each SCA1 clone sequence identified for the proband (II:3). ATXN1 CAG repeats were PCR amplified, cloned and sequenced. Mean allele sizes are 30 repeats and 38 repeats. [file Data_Sheet_1.zip › Figure 3/Panel B/65 Repeats Interrupted 1.png]

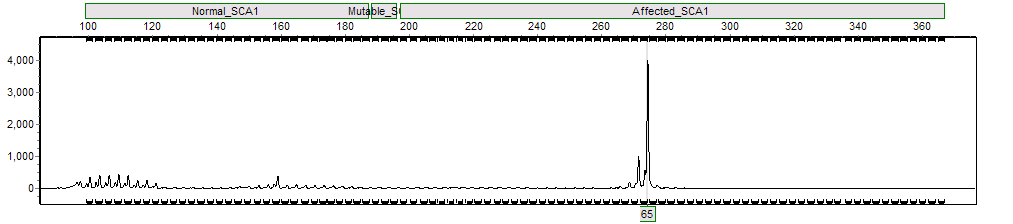

Supplement: TABLE S1 — Frequency of each SCA1 clone sequence identified for the proband (II:3). ATXN1 CAG repeats were PCR amplified, cloned and sequenced. Mean allele sizes are 30 repeats and 38 repeats. [file Data_Sheet_1.zip › Figure 3/Panel B/65 Repeats Interrupted 2.png]

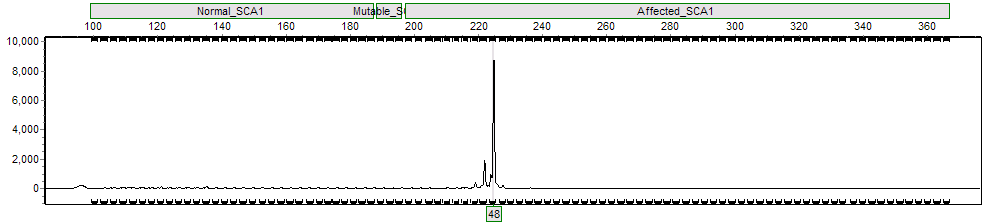

Supplement: TABLE S1 — Frequency of each SCA1 clone sequence identified for the proband (II:3). ATXN1 CAG repeats were PCR amplified, cloned and sequenced. Mean allele sizes are 30 repeats and 38 repeats. [file Data_Sheet_1.zip › Figure 3/Panel D/48 Repeats Interrupted.png]

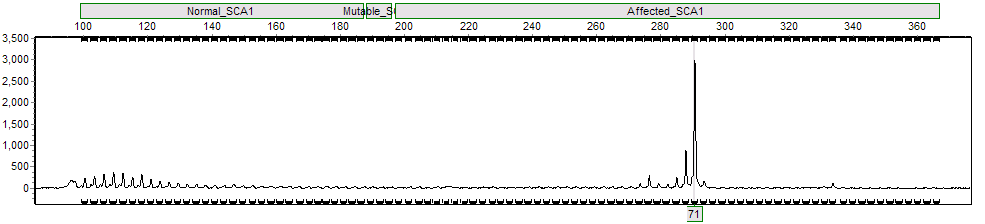

Supplement: TABLE S1 — Frequency of each SCA1 clone sequence identified for the proband (II:3). ATXN1 CAG repeats were PCR amplified, cloned and sequenced. Mean allele sizes are 30 repeats and 38 repeats. [file Data_Sheet_1.zip › Figure 3/Panel C/71 Repeats Interrupted.png]

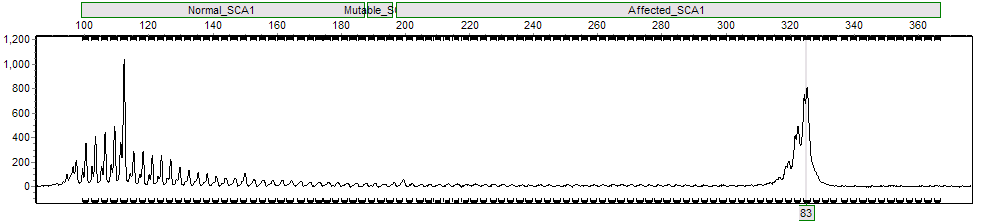

Supplement: TABLE S1 — Frequency of each SCA1 clone sequence identified for the proband (II:3). ATXN1 CAG repeats were PCR amplified, cloned and sequenced. Mean allele sizes are 30 repeats and 38 repeats. [file Data_Sheet_1.zip › Figure 3/Panel C/83 Repeats Interrupted.png]

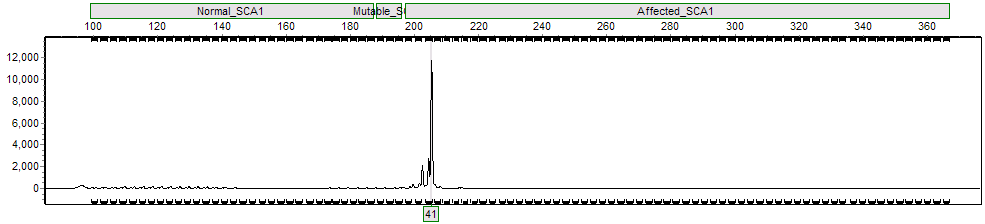

Supplement: TABLE S1 — Frequency of each SCA1 clone sequence identified for the proband (II:3). ATXN1 CAG repeats were PCR amplified, cloned and sequenced. Mean allele sizes are 30 repeats and 38 repeats. [file Data_Sheet_1.zip › Figure 3/Panel A/41 Repeats Interrupted.png]

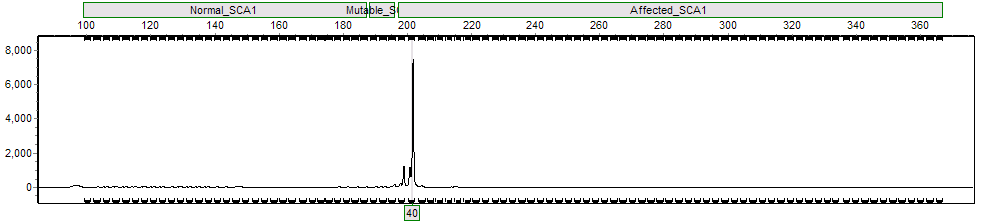

Supplement: TABLE S1 — Frequency of each SCA1 clone sequence identified for the proband (II:3). ATXN1 CAG repeats were PCR amplified, cloned and sequenced. Mean allele sizes are 30 repeats and 38 repeats. [file Data_Sheet_1.zip › Figure 3/Panel A/40 Repeats Uninterrupted.png]

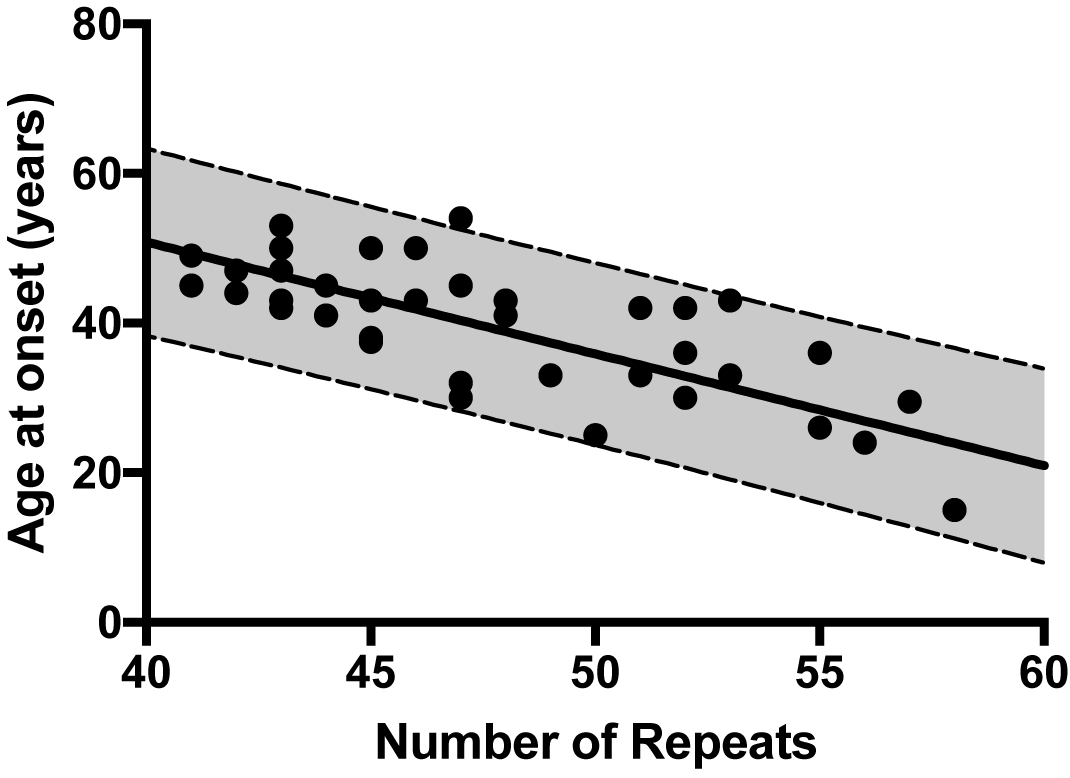

Supplement: DATASHEET S1 — Individual fragment analysis traces as shown in Figure 3. [file Image_1.tif]
